# Relationships among Depression, Anxiety, Sleep, and Quality of Life in Patients with Parkinson's Disease in Taiwan

**DOI:** 10.1155/2016/4040185

**Published:** 2016-05-15

**Authors:** Jun-Yu Fan, Bao-Luen Chang, Yih-Ru Wu

**Affiliations:** ^1^Department of Nursing, Chang Gung University of Science and Technology, No. 261, Wenhua 1st RD, Guishan District, Taoyuan 33303, Taiwan; ^2^Department of Nursing, Linkou Chang Gung Memorial Hospital, No. 5, Fu-Hsing Street, Guishan District, Taoyuan 33303, Taiwan; ^3^Department of Neurology, Linkou Chang Gung Memorial Hospital and Chang Gung University College of Medicine, No. 5, Fu-Hsing Street, Guishan District, Taoyuan 33303, Taiwan

## Abstract

The aim of this study was to examine the relationships among depression, anxiety, sleep disturbances, Parkinson's disease (PD) symptoms, PD medications, and health-related quality of life (QOL) and to identify the predictors of health-related QOL in PD patients. To do this, we administered a battery of questionnaires and rating scales (validated Chinese versions), including the Unified Parkinson's Disease Rating Scale, 39-item Parkinson's Disease Questionnaire, Parkinson's Disease Sleep Scale-2, Beck Depression Inventory, and Beck Anxiety Inventory, to 134 patients with PD whose Minimental State Examination scores were ≥24. We found that patients who reported having poorer QOL had longer disease durations, more severe PD symptoms, higher Hoehn and Yahr stages, and higher levodopa dosages, as well as higher levels of anxiety and depression, more sleep disturbances, and poorer overall cognitive statuses. Among these variables, the cognitive status, dependency of activities of daily living, depression, and anxiety were identified as predictors of QOL in PD patients and were all significant and independent factors of poor QOL in PD patients. The clinicians should be aware of the effects of these factors on QOL and attempt to treat comorbid psychiatric conditions to improve the PD patients' QOL.

## 1. Introduction

Parkinson's disease (PD) is primarily considered to be a movement disorder, with a prevalence of about 1% in individuals over 60 years of age [1]. Dopamine replacement therapy, which has been the dominant treatment for PD since the early 1960s, effectively relieves the motor deficits [2]. Moreover, systematic reviews have indicated that various nonmotor symptoms, including psychiatric and behavioral problems, usually do not respond to dopaminergic medications and thus are major factors that can affect the patients' health-related quality of life (QOL), progression of disability, level of care dependency, and the caregivers' level of distress [3].

Since PD is a chronic and progressive neurological condition, preserving the patients' QOL is important to both the patients and the healthcare providers. Previous studies have shown that the prevalence of nonmotor symptoms such as depression, anxiety, and sleep disturbances is higher in patients with PD than in the general population [4–12]. Indeed, depression and anxiety are common and frequently coexist in patients with PD; moreover, they seem to be associated with the patients' QOL [10]. In fact, the nonmotor symptoms of PD may have a greater effect on the QOL of patients with PD than the motor symptoms. However, many of the previous studies on PD have described the effects of depression on QOL rather than the effects of anxiety, even though anxiety can contribute to a patient's discomfort. In addition to depression and anxiety, sleep disturbances, such as poor sleep quality, excessive daytime sleepiness, delays in falling asleep, and difficulty staying asleep, have been estimated to occur in ~60–98% of patients with PD [8, 9]. Currently, it is unclear whether these nonmotor symptoms share common pathophysiological mechanisms [10]. However, some studies have shown that mood disorders are negatively correlated with sleep disturbances in patients with PD [13, 14].

Past studies mainly focused on the associations among the motor symptoms and one or two nonmotor symptoms. Few studies have investigated the impact of depression, anxiety, sleep disturbances, cognitive status, PD symptoms, and PD medications on the patients' health-related QOL [4–14]. Therefore, the aim of the present study was to examine the relationships among depression, anxiety, sleep disturbances, PD symptoms, PD medications, and health-related QOL and to identify the predictors of health-related QOL in patients with PD.

## 2. Materials and Methods

### 2.1. Subjects

Patients from the outpatient clinics of a movement disorder specialist (Yih-Ru Wu) who met the inclusion criteria were enrolled in this study. The inclusion criteria were as follows: (1) meeting the UK Parkinson's Disease Society Brain Criteria [15]; (2) age between 30 and 90 years; (3) Minimental State Examination (MMSE, Chinese version) score ≥24; and (4) regular follow-up visits every 1–3 month(s) for the current study at the medical center. Exclusion criteria were as follows: (1) secondary Parkinsonism due to an organic disorder or traumatic brain injury condition and (2) participants without a confirmed PD diagnosis and with irregular follow-up visits, those who were disorientated or unable to follow instructions, or those with other genetic or degenerative diseases.

This cross-sectional study was approved by the institutional review board of the Chang Gung Memorial Hospital Ethics Committee (number 101-2491B). After written informed consent was obtained, patients underwent a structured interview during which we obtained information on their global cognitive function, QOL, sleep quality, and emotional status. Each patient's levodopa equivalent daily dosage (LEDD) was calculated according to the described formula [16]. A trained research assistant conducted the interviews, each of which lasted around 60 min and was held immediately after the follow-up clinic. A total of 226 patients with a clinical diagnosis of PD participated in the study from March to December 2013. Of these, 58 were excluded for not meeting the inclusion criteria and 17 were excluded due to incomplete data; thus, the final sample consisted of 134 patients with PD. As shown in Table 1, most of the participants were men (85, 63.4%) and the mean age and disease onset age of the study cohort were 64.98 ± 9.19 years and 57.29 ± 9.19 years, respectively. More than half of the participants were ≥65 years old (77, 57.46%). Less than half of the participants were still working either part-time or full-time jobs (65, 48.51%), and of those still working, 30 (22.39%) were ≥65 years old.

### 2.2. Instruments

The MMSE was used as a brief screening tool for cognitive impairment [17]; the disease stage and symptoms were evaluated using the Hoehn and Yahr scale (H&Y) [18] and the Unified Parkinson's Disease Rating Scale (UPDRS) [19], respectively. The patients' QOL and sleep quality were assessed using the 39-item Parkinson's Disease Questionnaire (PDQ-39) [20] and Parkinson's Disease Sleep Scale-2 (PDSS-2) [21], respectively, which were each answered on a five-point Likert scale ranging from 0 (never) to 4 (always), with higher scores indicating poorer QOL and sleep quality, respectively. Since comorbidity of anxiety and depression can be a concern, the self-reported Beck Depression Inventory (BDI) [22] and Beck Anxiety Inventory (BAI) [23] were utilized to detect depression and anxiety, respectively. Both the BDI and BAI were answered on a four-point Likert scale ranging from 0 (not at all) to 3 (severely), with higher scores indicating higher levels of anxiety and depression [22, 23], respectively. All of the instruments we used were the validated Chinese versions.

### 2.3. Statistical Analysis

The demographical factors and characteristics of the study participants are presented as frequencies and percentages for categorical variables or as the mean and standard deviation for continuous variables. Participants' characteristics and variables of interest among the different depression/anxiety severity indexes were compared by using Chi-squared tests with Fisher's exact tests and one-way analyses of variance (ANOVA) followed by Bonferroni post hoc multiple comparisons tests. The relationships among participants' characteristics and other variables of interest were examined using Pearson's correlation coefficient. An multiple regression analysis (forward) was employed to assess the potential predictive variables for QOL in patients with PD. Since severe multicollinearity among independent variables may reduce the variance estimated in the regression model, prior to exploring the predictors, a multicollinearity diagnosis using variance inflation factors of less than 10 was conducted to examine whether the potential regression model violated the assumption of the regression model. Data analyses were conducted using SPSS 22 (IBM SPSS Inc., Chicago, IL) and the significance level was set at *p* < 0.05. All tests were two-tailed.

## 3. Results

### 3.1. Participants' Disease-Related Characteristics

Table 1 displays the characteristics of the study participants. In terms of the disease-related attributes, the mean disease duration (years), MMSE score, and H&Y stage were 7.86 ± 5.55 years, 27.41 ± 1.81, and 1.43 ± 0.63, respectively. Sixty-four (47.8%) and 70 (52.2%) participants had MMSE scores above 27 (normal cognition) and within 24 to 27 (mild cognitive impairment), respectively. Among the participants, 108 (80.6%) were classified as having mild PD, with H&Y stages of 1–1.5. The mean UPDRS score was 39.48 ± 18.30 and the mean UPDRS part I (mentation, behavior, and mood), part II (activities of daily living (ADL)), part III (motor evaluation), and part VI (therapy complications) scores were 3.36 ± 2.32, 12.93 ± 6.30, 20.29 ± 11.03, and 3.50 ± 3.22, respectively. The mean total PDSS-2 score, overall PDQ-39 score, and LEDD were 18.36 ± 16.92, 37.99 ± 25.40, and 617.06 ± 454.73, respectively.

The severities of depression and anxiety were based on the cut-off values of 13 and 7, respectively, on the BDI and BAI questionnaires [22, 23], and patients with PD were classified into the following groups according to their depression and anxiety scores, respectively: normal (0–13 versus 0–7), mild (14–19 versus 8–15), moderate (20–28 versus 16–25), and severe (29–63 versus 26–63). There were more participants (92, 68.70%) with different levels of anxiety compared to depression (49, 36.60%) in this sample. To demonstrate that anxiety coexisted with depression in patients with PD, a 4 × 4 cross table was conducted. The results showed that 39 of the 134 participants (29.10%) had neither depression nor anxiety. Anxiety in the absence of depression was evident in 46 patients (34.33%), depression in the absence of anxiety was evident in three patients (2.22%), and anxiety coexisting with depression was observed in 46 patients (34.33%).  Table 2 displays the levels of coexistence for anxiety and depression among the study participants.

As for whether the participants' characteristics showed sex differences, we found that women with PD demonstrated significantly higher scores (more depression/anxiety symptoms) on the BDI (*t*(132) = −2.28, *p* = 0.024) and BAI (*t*(132) = −3.17, *p* = 0.004) and higher scores (poorer QOL) on the PDQ-39 (*t*(132) = −2.08, *p* = 0.039) than men with PD. Moreover, the Chi-squared test with Fisher's exact test, which was used to examine sex differences in the abovementioned four anxiety groups, as well as in the four depression groups, revealed that there was a higher proportion of women in the depression groups (*χ* ^2^(3) = 12.46, *p* = 0.005) but a higher proportion of men in the anxiety groups (*χ* ^2^(3) = 13.61, *p* = 0.003).

The one-way ANOVA for examining the differences in the characteristics among the four anxiety groups showed that there were significant differences in the disease duration (*F*(1,130) = 3.41, *p* = 0.020) and LEDD (*F*(1,130) = 4.39, *p* = 0.006), as well as in the UPDRS (*F*(1,130) = 8.72, *p* < 0.001), UPDRS part I (*F*(1,130) = 8.07, *p* < 0.001), UPDRS part II (*F*(1,130) = 10.95, *p* < 0.001), UPDRS part VI (*F*(1,130) = 10.33, *p* < 0.001), BDI (*F*(1,130) = 25.02, *p* < 0.001), PDQ-39 (*F*(1,130) = 28.02, *p* < 0.001), and PDSS-2 (*F*(1,130) = 10.84, *p* < 0.001) scores. Similar results were observed for the four depression groups, as significant differences were noted in the disease duration (*F*(1,130) = 9.27, *p* < 0.001) and LEDD (*F*(1,130) = 8.90, *p* < 0.001), as well as in the UPDRS (*F*(1,130) = 10.09, *p* < 0.001), UPDRS part I (*F*(1,130) = 7.52, *p* < 0.001), UPDRS part II (*F*(1,130) = 12.73, *p* < 0.001), UPDRS part VI (*F*(1,130) = 16.25, *p* < 0.001), BDI (*F*(1,130) = 21.66, *p* < 0.001), PDQ-39 (*F*(1,130) = 36.71, *p* < 0.001), and PDSS-2 (*F*(1,130) = 11.14, *p* < 0.001) scores. Post hoc Bonferroni tests were performed to examine the differences in the disease duration; UPDRS; UPDRS parts I, II, and VI; BDI; PDQ-39; PDSS-2; and LEDD among the four depression groups and four anxiety groups.  Table 3 shows the differences in the above variables among the four depression groups and four anxiety groups.

### 3.2. Correlations Analyses

The results of the correlation analyses showed that the PDQ-39 scores were significantly and positively correlated with the disease duration (*r* = 0.35, *p* < 0.01), LEDD (*r* = 0.35, *p* < 0.01), and H&Y stage (*r* = 0.31, *p* < 0.01), as well as with the UPDRS (*r* = 0.58, *p* < 0.01), UPDRS part I (*r* = 0.46, *p* < 0.01), UPDRS part II (*r* = 0.62, *p* < 0.01), UPDRS part III (*r* = 0.38, *p* < 0.01), UPDRS part VI (*r* = 0.42, *p* < 0.01), BAI (*r* = 0.65, *p* < 0.01), BDI(*r* = 0.68, *p* < 0.01), and PDSS-2 (*r* = 0.38, *p* < 0.01) scores. The PDQ-39 score was inversely related to the overall cognitive status (MMSE score) (*r* = −0.24, *p* < 0.01). Thus, participants with longer disease durations, higher PD severity stages, more PD symptoms, poorer mood, higher dependency of ADL, more motor impairments, more therapy-related complications, more depression and anxiety symptoms, poorer sleep quality, higher LEDDs, and lower cognitive statuses demonstrated poorer QOL.  Table 4 lists the interrelationships among these variables.

### 3.3. Regression Analyses

All of the variance inflation factors were less than 10 (range: 1.36 to 3.56), indicating that the potential regression model did not violate the assumption of the regression model. The PDQ-39 scores were regressed against various potential predictors including sex, disease duration, H&Y stage, LEDD, and scores on the UPDRS parts I to VI, BAI, BDI, and PDSS-2. The overall model reached statistical significance (*F*(13,120) = 21.05, *p* < 0.001, and *R* ^2^ = 0.70 (adjusted *R* ^2^ = 0.66)). The MMSE score had a significant effect on the QOL of patients with PD (*B* = −1.97, *p* = 0.019, and 95% confidence interval (CI) = −3.60–0.33), after adjusting for the rest of the 11 predictors (covariates); that is, the lower the cognitive function, the poorer the reported QOL. After adjusting for the rest of the 11 covariates, the UPDRS part II (the level of dependency of ADL) scores had a significant effect on the QOL of patients with PD (*B* = 1.47, *p* < 0.001, and 95% CI = 0.72–0.22); that is, the higher the level of dependency, the lower the reported QOL. In addition, the BDI scores had a significant effect on the QOL of patients with PD (*B* = 0.95, *p* < 0.001, and 95% CI = 0.60–1.30), after adjusting for the remaining 11 predictors (covariates); that is, the higher the level of depression reported, the poorer the QOL reported. Similarly, after adjusting for the remaining 11 covariates, we found that the BAI scores had a significant effect on the QOL of patients with PD (*B* = 0.70, *p* = 0.001, and 95% CI = 0.29–1.10); that is, the higher the level of anxiety reported, the poorer the QOL reported.

In summary, the MMSE, UPDRS part II (the level of dependency of ADL), depression, and anxiety scores were significant predictors of QOL in patients with PD. The proportion of variance (*R* ^2^) explained by the regression model amounted to 70.00% (adjusted 66.20%).  Table 5 shows the predictors of the PDQ-39.

## 4. Discussion

Our study demonstrated that PD patients who reported poorer QOL also had longer disease durations, more severe PD symptoms, higher Hoehn and Yahr stages, and higher levodopa dosages, as well as higher levels of anxiety and depression, more sleep disturbances, and poorer overall cognitive statuses. The results also showed that the disease duration, severity of symptoms, and medication usage greatly influenced the QOL in patients with PD, which has been reported previously in the literature [24, 25].

Here, we found that women with PD were more likely to develop depression and anxiety and have higher BDI (22, 55.10%) and BAI (38, 77.55%) scores, respectively, compared to men (22, 25.88%, and 54, 63.53%, resp.). These results were consistent with the prevalence of these disorders in the general population, where women have a twofold chance of developing depressive and anxiety disorders compared to men [25, 26]; however, our findings were different from those of several other studies that reported no sex differences in the development of depression and anxiety in the PD population [27–29].

Regarding the presence of anxiety and depression, analysis showed that about one-third of the participants had neither depression nor anxiety. Depression alone was only noted in 2.2% of patients. Anxiety coexisted with depression in about 34.33% of patients, which is lower than the results reported by Pontone et al. [30] (55%) and Yamanishi et al. [10] (41%). Anxiety in the absence of depression was evident in about 30% of the patients in the present study, and similar findings were reported by Richard [31] and Yamanishi et al. [10]. In our study, more participants had anxiety compared to depression. Indeed, compared to previous studies, which typically reported anxiety in 20–46% of patients with PD [10], our study showed a higher prevalence of anxiety (68.7%). The reasons for this difference may include (1) utilization of different assessment scales in different cohort studies, (2) reaction to disability of PD in different populations, and (3) neurochemical changes due to the disease itself, which further complicate the diagnosis of anxiety in patients with PD [32]. Despite these differences, our findings support the fact that both anxiety and depression are core features of PD.

In the present study, depression and anxiety were significantly associated with sleep disturbances and disease severity in nondemented patients with PD. Previous studies also found that mood disorders were important risk factors for poor sleep quality in patients with PD [13, 31]. In contrast to our results, Menza and Rosen [33] reported that depression and anxiety did not contribute significantly to any of the sleep quality variables. Additionally, Tse et al. [34] and Chaudhuri et al. [35] demonstrated that the mean total PDSS-2 score correlated with the H&Y stage. Our study also showed that poor sleep quality at night was significantly associated with the severity of PD without dementia.

In addition, we found that motor symptoms (UPDRS) and symptoms of anxiety and depression were major predictors of QOL in patients with PD, indicating that the nonmotor symptoms may play a more important role than the motor symptoms in a patient's QOL. These results are consistent with previous reports showing that neuropsychiatric comorbidities including depression, anxiety, psychosis, and apathy are of major concern and can contribute to a poor QOL once the patient's motor symptoms have been controlled with drugs [32, 34–36].

Strengths of the present study include a prospective design and an adequate number of patients. It is also important to mention that we used a more sensitive measure of PD motor symptoms (UPDRS) rather than only using the disease duration and H&Y stage. Additionally, we only recruited patients with adequate cognitive function, which increased the likelihood of accurate evaluations.

However, several limitations of our cross-sectional study should be mentioned. First, we used brief self-report scales for rating depression and anxiety. This prohibited us from clarifying the nature of the depressive and anxiety disorders in more detail and from determining whether the specific type of depression or anxiety disorder had a differential impact on the QOL. Second, this was only a cross-sectional study, and the direction of cause among the variables examined cannot be determined. Since depression and anxiety were identified as predictors of QOL in the current study, longitudinal studies would be beneficial for assessing the changes in the QOL in this population after administering treatment for depression and anxiety. Furthermore, replicating this study with a psychiatrist-based interview may help elucidate whether certain types of depression or anxiety disorders have greater effects on QOL and may clarify the frequency of major depressive disorder and general anxiety disorder in PD.

A better understanding of the factors that have the greatest effect on a patient's well-being is important for developing new management plans in PD. A comprehensive evaluation scheme of PD severity, which combines both motor and nonmotor assessments, may enhance the clinician's ability to address the features of PD more holistically, which in turn may affect the patient's QOL and disability. Screening for nonmotor symptoms is particularly important, as these symptoms seem to consistently have adverse effects on the functional status and health-related QOL of patients over the course of the disease. Future research should address this issue by including more participants and measuring their dispositional characteristics.

## Figures and Tables

**Table 1:** Characteristics of the participants (*N* = 134).

Characteristics	Mean (±SD) or *n* (%)	Range (min–max)
*Age* (years)	64.98 (±9.19)	41–87
*Sex*		
Male	85 (63.4%)	
Female	49 (36.6%)	
*Work*		
Yes	65 (48.51%)	
No	69 (51.49%)	
*Onset age* (years)	57.29 (±10.66)	29–85
*Disease duration* (years)	7.86 (±5.55)	0–23
*MMSE*	27.41 (±1.81)	24–30
*H*&*Y stage*	1.43 (±0.64)	1–4
1 & 1.5	108 (80.6%)	
2 & 2.5	20 (14.9%)	
3 & 4	6 (4.5%)	
*UPDRS*	39.48 (±18.30)	4–93
Part I	3.36 (2.32)	0–13
Part II	12.93 (6.3)	1–32
Part III	20.29 (11.03)	2–58
Part IV	3.50 (3.22)	0–18
*BAI*	12.23 (±18.30)	0–45
Normal (0–7)	42 (31.30%)	
Mild (8–15)	57 (42.50%)	
Moderate (16–25)	24 (17.90%)	
Severe (26–63)	11 (8.20%)	
*BDI*	12.69 (±9.76)	0–51
Normal (0–13)	85 (63.40%)	
Mild (14–19)	26 (19.40%)	
Moderate (20–28)	12 (9.00%)	
Severe (29–63)	11 (8.20%)	
*PDSS-2*	18.36 (±16.92)	1–72
*PDQ-39*	37.99 (±25.40)	0–135
*LEDD *(mg)	617.06 (±454.73)	0–2395

MMSE: Minimental State Examination; H&Y: Hoehn and Yahr scale; UPDRS: Unified Parkinson's Disease Rating Scale; BAI: Beck Anxiety Inventory; BDI: Beck Depression Inventory; PDSS-2: Parkinson's Disease Sleep Scale-2; PDQ-39: 39-item Parkinson's Disease Questionnaire; LEDD: levodopa equivalent daily dosage.

**Table 2:** Coexistence of anxiety and depression (*N* = 134).

	Severity level of anxiety *n* (%)				Subtotal
	Normal	Mild	Moderate	Severe	
Severity level of depression *n* (%)					
Normal	39 (29.10%)	38 (28.4%)	7 (5.22%)	1 (0.74%)	85 (63.43%)
Mild	2 (1.49%)	13 (9.70%)	8 (6.00%)	3 (2.24%)	26 (19.40%)
Moderate	0 (0.00%)	4 (3.00%)	3 (2.24%)	5 (3.73%)	12 (8.96%)
Severe	1 (0.74%)	2 (1.49%)	6 (4.48%)	2 (1.49%)	11 (8.21%)
Subtotal	42 (31.34%)	57 (42.54%)	24 (17.91%)	11 (8.21%)	134 (100.00%)

Beck Anxiety Inventory: normal (0–7), mild (8–15), moderate (16–25), and severe (26–63).

Beck Depression Inventory: normal (0–13), mild (14–19), moderate (20–28), and severe (29–63).

**Table 3:** Differences in the participants' characteristics according to the severity of depression and anxiety (*N* = 134).

		Depression severity				*p*	Anxiety severity				*p*
		Normal (1)	Mild (2)	Moderate (3)	Severe (4)		Normal (1)	Mild (2)	Moderate (3)	Severe (4)	
*Sex*	*n* (%)	85 (63.40)	26 (19.40)	12 (9.00)	11 (8.20)	*∗∗* ^a^	42 (31.30)	57 (42.50)	24 (17.90)	11 (8.20)	*∗∗* ^a^
Male	85 (63.4%)	63 (47.01)	12 (8.96)	4 (2.99)	6 (4.48)		31 (23.13)	41 (30.60)	9 (6.72)	4 (2.99)	
Female	49 (36.6%)	22 (16.42)	14 (10.44)	8 (5.79)	5 (3.73)		11 (8.21)	16 (11.94)	15 (11.19)	7 (5.22)	
*Onset age *	M ± SD	58.73 ± 9.78	53.23 ± 12.83	56.25 ± 9.79	56.91 ± 11.36		59.95 ± 8.94	56.16 ± 11.73	56.79 ± 10.35	54.09 ± 10.86	
*Current age*		64.64 ± 9.02	64.96 ± 11.03	66.67 ± 6.60	65.82±9.03		65.36 ± 8.88	64.19 ± 9.48	66.88 ± 9.31	63.45 ± 9.02	
*Disease duration*		6.18 ± 4.53	11.73 ± 5.87	10.42 ± 7.00	8.91 ± 4.97	*∗∗∗*	6.00 ± 4.82	8.04 ± 5.41	10.08 ± 5.51	9.36 ± 7.09	*∗*
Post hoc^b^		(2) > (1)^*∗∗∗*^; (3) > (1)^*∗*^					(3) > (1)^*∗*^				
*MMSE*		27.51 ± 1.80	27.42 ± 1.92	27.00 ± 1.71	27.09 ± 1.81		27.43 ± 1.77	27.70 ± 1.74	26.67 ± 2.01	27.45 ± 1.57	
*UPDRS*		33.73 ± 15.10	53.00 ± 19.22	44.33 ± 19.45	46.64 ± 18.93	*∗∗∗*	29.62 ± 12.94	40.81 ± 18.42	48.83 ± 18.10	49.82 ± 19.15	*∗∗∗*
Post hoc^b^		(2) > ^*∗∗∗*^					(4) > (1)^*∗∗*^; (3) > (1)^*∗∗∗*^; (2) > (1)^*∗∗*^				
*UPDRS part I *		2.72 ± 2.23	4.08 ± 1.96	4.42 ± 2.19	5.36 ± 2.11	*∗∗∗*	2.21 ± 1.73	3.47 ± 2.46	4.86 ± 2.33	3.72 ± 1.35	*∗∗∗*
Post hoc^b^		(2) > (1)^*∗*^; (4) > (1)^*∗*^					(2) > (1)^*∗*^; (3) > (1)^*∗*^				
*UPDRS part II *		10.75 ± 4.99	18.15 ± 6.44	14.50 ± 5.99	15.64 ± 7.98	*∗∗∗*	9.02 ± 4.52	13.70 ± 5.88	16.16 ± 7.33	16.82 ± 5.34	*∗∗∗*
Post hoc^b^		(2) > (1)^*∗*^; (4) > (1)^*∗*^					(2) > (1)^*∗*^; (3) > (1)^*∗*^; (4) > (1)^*∗*^				
*UPDRS part III *		18.88 ± 9.64	24.31 ± 13.00	20.67 ± 12.76	21.72 ± 11.11		16.64 ± 8.72	21.23 ± 11.54	23.29 ± 11.64	22.82 ± 12.66	
*UPDRS part VI *		2.31 ± 2.18	6.46 ± 4.52	4.75 ± 2.53	4.36 ± 1.57	*∗∗∗*	1.69 ± 1.98	3.82 ± 3.66	4.54 ± 1.89	6.45 ± 3.50	*∗∗∗*
Post hoc^b^		(2) > (1)^*∗*^; (3) > (1)^*∗*^					(2)>(1)^*∗*^; (3)>(1)^*∗*^; (4) > (1) (2)^*∗*^				
*H&Y stage*		1.28 ± 0.50	1.90 ± 0.93	1.54 ± 0.54	1.36 ± 0.39	*∗∗∗*	1.23 ± 0.39	1.45 ± 0.65	1.73 ± 0.86	1.45 ± 0.65	*∗*
Post hoc^b^		(2) > (1)^*∗∗∗*^					(3)>(1)^*∗*^				
*PDSS-2*		13.94 ± 10.83	26.15 ± 12.72	25.33 ± 14.31	26.45 ± 12.32	*∗∗∗*	12.52 ± 11.11	17.21 ± 10.22	25.67 ± 16.75	30.64 ± 6.42	*∗∗∗*
*PDQ-39*		25.80 ± 16.63	54.85 ± 21.50	51.75 ± 13.64	77.36 ± 31.01	*∗∗∗*	18.43 ± 11.55	38.82 ± 17.94	57.71 ± 29.62	65.36 ± 29.07	*∗∗∗*
Post hoc^b^		(4) > (3)^*∗∗*^; (4) > (2)^*∗∗*^; (4) > (1)^*∗∗∗*^					(4) > (1)^*∗∗∗*^; (4) > (2)^*∗∗*^; (3) > (1)^*∗∗∗*^; (3) > (2)^*∗∗*^; (2) > (1)^*∗∗∗*^				
*LEDD *(mg)		489.95 ± 356.94	882.04 ± 481.16	940.26 ± 707.01	620.43 ± 325.62	*∗∗∗*	413.31 ± 323.94	638.47 ± 409.57	681.27 ± 298.08	728.86 ± 502.46	*∗∗∗*
Post hoc^b^		(3) > (1)^*∗∗*^; (2) > (1)^*∗∗∗*^					(3) > (1)^*∗*^; (2) > (1)^*∗*^				

M: mean; SD: standard deviation; MMSE: Mini-Mental State Examination; H&Y: Hoehn and Yahr scale; UPDRS: Unified Parkinson's Disease Rating Scale; BAI: Beck Anxiety Inventory; BDI: Beck Depression Inventory; PDSS-2: Parkinson's Disease Sleep Scale-2; PDQ-39: 39-item Parkinson's Disease Questionnaire; LEDD: levodopa equivalent daily dosage. ^a^Chi-squared test with Fisher's exact test; ^b^Bonferroni post hoc test; ^*∗*^ *p* < 0.05, ^*∗∗*^ *p* < 0.01, ^*∗∗∗*^ *p* < 0.001.

**Table 4:** The correlations among variables (*N* = 134).

	MMSE	Disease duration	H&Y stage	UPDRS	UPDRS part I	UPDRS part II	UPDRS part III	UPDRS part VI	BAI	BDI	PDSS-2	LEDD	PDQ-39
MMSE	—												
Disease duration	−0.02	—											
H&Y stage	−0.15	0.34^*∗∗*^	—										
UPDRS	−0.17	0.53^*∗∗*^	0.59^*∗∗*^	—									
UPDRS part I	−0.15	0.29^*∗∗*^	0.35^*∗∗*^	0.39^*∗∗*^	—								
UPDRS part II	−0.13	0.52^*∗∗*^	0.55^*∗∗*^	0.88^*∗∗*^	0.41^*∗∗*^	—							
UPDRS part III	−0.22^*∗∗*^	0.32^*∗∗*^	0.51^*∗∗*^	0.87^*∗∗*^	0.33^*∗∗*^	0.68^*∗∗*^	—						
UPDRS part VI	0.23^*∗∗*^	0.46^*∗∗*^	0.18^*∗*^	0.48^*∗∗*^	0.23^*∗∗*^	0.45^*∗∗*^	0.17	—					
BAI	−0.03	0.26^*∗∗*^	0.20^*∗*^	0.40^*∗∗*^	0.35^*∗∗*^	0.44^*∗∗*^	0.20^*∗*^	0.47^*∗∗*^	—				
BDI	−0.13	0.30^*∗∗*^	0.21^*∗*^	0.34^*∗∗*^	0.44^*∗∗*^	0.36^*∗∗*^	0.15	0.35^*∗∗*^	0.58^*∗∗*^	—			
PDSS-2	0.05	0.32^*∗∗*^	0.39^*∗∗*^	0.44^*∗∗*^	0.16	0.47^*∗∗*^	0.27^*∗∗*^	0.39^*∗∗*^	0.43^*∗∗*^	0.39^*∗∗*^	—		
LEDD	0.01	0.56^*∗∗*^	0.42^*∗∗*^	0.56^*∗∗*^	0.29^*∗∗*^	0.59^*∗∗*^	0.34^*∗∗*^	0.48^*∗∗*^	0.28^*∗∗*^	0.27^*∗∗*^	0.40^*∗∗*^	—	
PDQ-39	−0.24^*∗∗*^	0.35^*∗∗*^	0.31^*∗∗*^	0.58^*∗∗*^	0.46^*∗∗*^	0.62^*∗∗*^	0.38^*∗∗*^	0.42^*∗∗*^	0.65^*∗∗*^	0.68^*∗∗*^	0.38^*∗∗*^	0.35^*∗∗*^	—

H&Y: Hoehn and Yahr scale; UPDRS: Unified Parkinson's Disease Rating Scale; BAI: Beck Anxiety Inventory; BDI: Beck Depression Inventory; PDSS-2: Parkinson's Disease Sleep Scale-2; PDQ-39: 39-item Parkinson's Disease Questionnaire; LEDD: levodopa equivalent daily dosage. ^*∗*^ *p* < 0.05 and ^*∗∗*^ *p* < 0.01.

**Table 5:** Predictors of PDQ-39 in the forward multiple regression analysis (*N* = 134).

Model	Unstandardized coefficients		
	*B*	SE	*p* value
Constant	53.20	27.70	
Sex			
Males versus females	−3.28	3.16	0.301
Current age (years)	0.04	0.16	0.816
Disease duration (years)	−0.10	0.30	0.744
H&Y stage (1–4)	−2.26	2.79	0.421
LEDD (mg)	−0.002	0.004	0.643
MMSE	−1.97	0.827	0.019
UPDRS part I	0.53	0.66	0.432
UPDRS part II	1.47	0.38	<0.001
UPDRS part III	0.08	0.17	0.652
UPDRS part IV	0.66	0.56	0.246
BDI	0.70	0.21	0.001
BAI	0.95	0.18	<0.001
PDSS-2	−0.10	0.13	0.445

	*R* ^2^	0.70	

	Adjusted *R* ^2^	0.66	

*B*: unstandardized regression coefficient; SE: standard error; H&Y: Hoehn and Yahr scale; UPDRS: Unified Parkinson's Disease Rating Scale; BAI: Beck Anxiety Inventory; BDI: Beck Depression Inventory; PDSS-2: Parkinson's Disease Sleep Scale-2; PDQ-39: 39-item Parkinson's Disease Questionnaire; LEDD: levodopa equivalent daily dosage.
